# Characterization and High-Level Periplasmic Expression of Thermostable α-Carbonic Anhydrase from *Thermosulfurimonas Dismutans* in *Escherichia Coli* for CO_2_ Capture and Utilization

**DOI:** 10.3390/ijms21010103

**Published:** 2019-12-22

**Authors:** Byung Hoon Jo, In Seong Hwang

**Affiliations:** 1 Division of Life Science and Research Institute of Life Science, Gyeongsang National University, Jinju 52828, Korea; 2 Division of Applied Life Science (BK21 Plus), Gyeongsang National University, Jinju 52828, Korea

**Keywords:** carbonic anhydrase, *Thermosulfurimonas dismutans*, thermostability, periplasmic expression, carbon capture, whole-cell biocatalyst

## Abstract

Carbonic anhydrase (CA) is a diffusion-controlled enzyme that rapidly catalyzes carbon dioxide (CO_2_) hydration. CA has been considered as a powerful and green catalyst for bioinspired CO_2_ capture and utilization (CCU). For successful industrial applications, it is necessary to expand the pool of thermostable CAs to meet the stability requirement under various operational conditions. In addition, high-level expression of thermostable CA is desirable for the economical production of the enzyme. In this study, a thermostable CA (*td*CA) of *Thermosulfurimonas dismutans* isolated from a deep-sea hydrothermal vent was expressed in *Escherichia coli* and characterized in terms of expression level, solubility, activity and stability. *td*CA showed higher solubility, activity, and stability compared to those of CA from *Thermovibrio ammonificans*, one of the most thermostable CAs, under low-salt aqueous conditions. *td*CA was engineered for high-level expression by the introduction of a point mutation and periplasmic expression via the Sec-dependent pathway. The combined strategy resulted in a variant showing at least an 8.3-fold higher expression level compared to that of wild-type *td*CA. The *E. coli* cells with the periplasmic *td*CA variant were also investigated as an ultra-efficient whole-cell biocatalyst. The engineered bacterium displayed an 11.9-fold higher activity compared to that of the recently reported system with a halophilic CA. Collectively these results demonstrate that the highly expressed periplasmic *td*CA variant, either in an isolated form or within a whole-cell platform, is a promising biocatalyst with high activity and stability for CCU applications.

## 1. Introduction

Carbonic anhydrase (CA) is a zinc-metalloenzyme that catalyzes carbon dioxide (CO_2_) hydration: CO_2_ + H_2_O → HCO_3_^−^ + H^+^ [1]. CAs are widespread in the three domains of life and are classified into seven distinct families (α, β, γ, δ, ζ, η and θ), performing important physiological roles in various organisms from microbes to human [1]. CAs show diffusion-controlled kinetics with a *k*_cat_ of up to 4.4 × 10^6^ s^−1^ [2]. This feature makes them powerful and eco-friendly catalysts for bioinspired CO_2_ capture and utilization (CCU) which is one of the promising routes to the mitigation of greenhouse gas emissions. The hydration of CO_2_, which is the rate-determining step of CO_2_ capture into HCO_3_^−^, can be accelerated by the catalysis of CA [3]. The rapid formation of HCO_3_^−^ can benefit CCU processes that utilize HCO_3_^−^ as a feedstock for mineral carbonation [4,5], production of value-added chemicals [6,7], or cultivation of photoautotrophic microorganisms [8] by accelerating the reactions and reducing energy requirement, thus improving the efficiencies of CCU. In many cases, the primary barriers to the industrial application of CA are the low stability of the enzymes and the relatively high enzyme production cost.

Bioprospecting novel CA or engineering existing CA has been performed to obtain thermostable CA. CAs from thermophiles, halophiles, and alkaliphiles have been searched and examined for their potential industrial applicability [9,10,11,12,13,14,15]. Some researchers have shown that the stability of CA can be improved by protein engineering via rational design [16,17,18,19,20] or directed evolution [21]. However, the stabilities of CAs were not sufficiently high in most cases, and high stabilities were achieved only under specific test conditions such as high-salt condition [12,18] or organic solvent [21]. Moreover, studies on the high-level expression of recombinant CA have been rarely conducted in spite of its importance for the economic enzyme production [5,22]. Thus, expanding the pool of stable CAs and improving the expression level are prerequisite tasks to meet the enzyme properties required under various operational conditions encountered in various CCU applications and to increase the economic feasibility of using CA as a CO_2_ capture-promoting catalyst.

Enzymes from deep-sea hydrothermal vents are adapted to extreme environmental conditions, making them potential biocatalysts for industrial applications [23]. *Thermosulfurimonas dismutans* isolated from a deep-sea hydrothermal vent is an extremely thermophilic bacterium utilizing CO_2_ as the sole carbon source [24]. The maximal growth temperature of *T. dismutans* is 92 °C, which is much higher than that (80 °C) of *Thermovibrio ammonifican*s whose α-CA (*ta*CA) is well characterized for its high thermostability [9,25,26]. Considering the important role of α-CA as the initial enzyme for the autotrophic CO_2_ metabolism [27,28], α-CA (*td*CA) of *T. dismutans* is expected to be highly active and stable.

In the present work, we expressed and purified recombinant *td*CA in *Escherichia coli* and performed a comparative study of *td*CA and *ta*CA on expression level, solubility, activity, and stability that are important factors for industrial applications of CA. *td*CA was further engineered for high-level expression by the introduction of a point mutation and periplasmic expression via the Sec-dependent pathway. Finally, the engineered bacterium with the periplasmic *td*CA was investigated as an ultra-efficient whole-cell biocatalyst for CCU.

## 2. Results and Discussion

### 2.1. Comparative Analysis of Bacterial Thermostable α-CA Sequences

**Figure 1:**
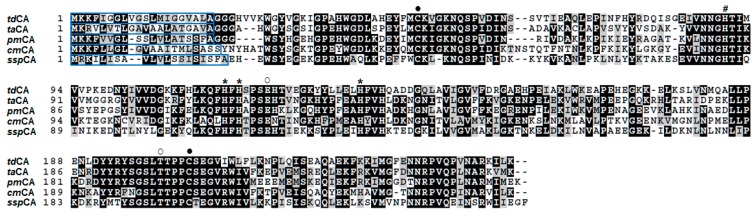
Multiple alignment of representative thermophilic α-CAs. The sequences are from *T. dismutans* (*td*CA; OAQ21602), *T. ammonificans* (*ta*CA; WP_013538320), *P. marina* (*pm*CA; ACO04804), *C. mediatlanticus* (*cm*CA; EDM23829), and *S. yellowstonense* (*ssp*CA; ACD66216). Conserved or similar residues across three or more sequences are shaded in black or gray, respectively. The native signal sequences are enclosed in blue boxes. The two cysteine residues for the formation of the intramolecular disulfide bond are indicated by a closed circle (●). The three zinc ligand histidine residues (*) and proton shuttling histidine residue (#) are marked. The two gate-keeper residues are also indicated by an open circle (○).

The amino acid sequences of *td*CA and *ta*CA were aligned along with those of other α-CAs from the selected Gram-negative thermophiles, *Persephonella marina* (*pm*CA), *Caminibacter mediatlanticus* (*cm*CA) and *Sulfurihydrogenibium yellowstonense* (*ssp*CA) (Figure 1). The residues highly conserved across bacterial α-CAs are also present in *td*CA including three zinc ligand (His-115, His-117, and His-134; *td*CA numbering system), proton shuttle residue (His-90), two gate-keeper residues (Glu-121 and Thr-200) and two cysteine residues forming an intramolecular disulfide bond (Cys-49 and Cys-204) [29]. All of the selected CAs have an N-terminal signal peptide, suggesting the periplasmic localization in the original hosts. The average sequence identity across the five sequences is 49.9%, while the highest identity is found between the sequences of *ta*CA and *pm*CA (62.0%). The sequence of *td*CA shows the highest and the lowest identities with those of *ta*CA (56.3%) and *ssp*CA (44.4%), respectively, implying that *td*CA is more closely related to *ta*CA than the other thermophilic CAs.

### 2.2. Expression and Purification of Recombinant CAs

**Figure 2:**
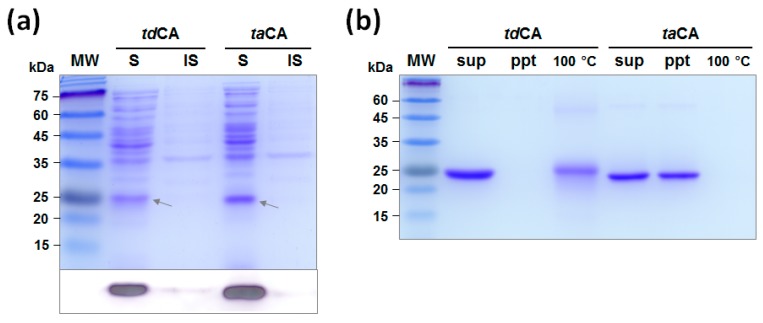
Expression and purification of recombinant carbonic anhydrases (CAs). (**a**) Expression of *td*CA and *ta*CA analyzed by SDS-PAGE, followed by Coomassie blue staining (upper) and Western blotting using an anti-His_6_ antibody (lower). Cell lysates were fractionated into soluble and insoluble fractions, and they were separately loaded. The arrow indicates the band position of each CA. (**b**) Purified recombinant CAs and their solubility analyzed by SDS-PAGE, followed by Coomassie blue staining. Lanes: MW, molecular mass marker; S, soluble fraction; IS, insoluble fraction; sup, supernatant; ppt, precipitated pellet; 100 °C, supernatant after boiling.

The genes for *td*CA and *ta*CA were expressed in *E. coli* BL21(DE3) using the strong T7*lac* promoter system. The hexahistidine (His_6_)-tagged recombinant CAs without the predicted signal peptides were expressed and accumulated in the cytoplasm of *E. coli*. Both recombinant CAs were successfully produced in soluble forms with the estimated molecular weights of 27.2 kDa and 27.0 kDa for *td*CA and *ta*CA, respectively, which correspond to the band positions in the protein gel (Figure 2a). The expression level of *td*CA was 20% lower than that of the *ta*CA in *E. coli* BL21(DE3) strain, as revealed by the densitometric analysis of soluble CAs on the Western blot result (Figure 2a).

**Figure 3:**
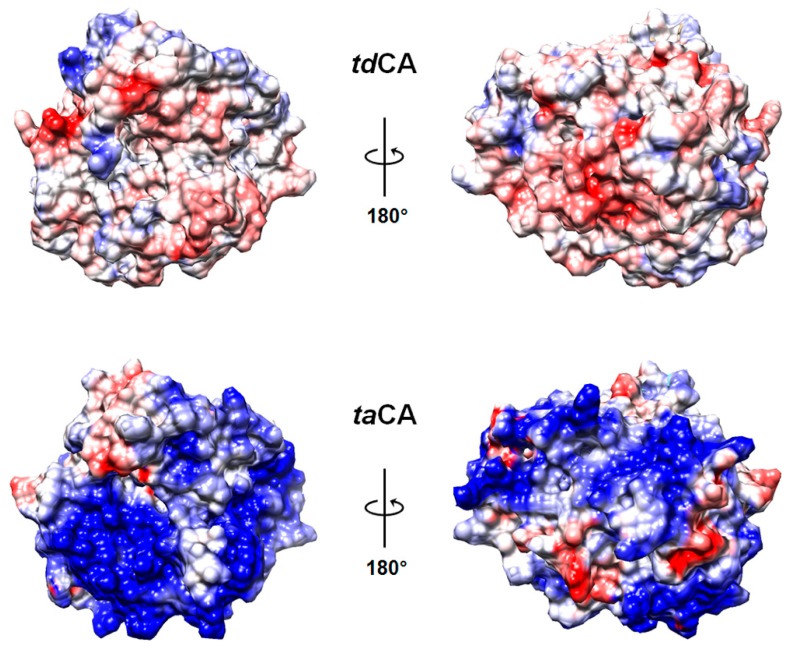
Electrostatic surface potential of CA. Electrostatic surface potentials of *td*CA and *ta*CA (protein data bank (PDB) ID: 4C3T) are represented on a scale from − 10 kT/e (red) to + 10 kT/e (blue). The predicted structure of *td*CA was first visualized in an orientation showing the active-site cavity in the center of the structure, and the structure of *ta*CA was then aligned according to the orientation of *td*CA. Only one (chain A) of the two chains was taken for the visualization of *ta*CA.

When the recombinant enzymes were purified by His_6_-tag affinity chromatography and dialyzed against sodium phosphate buffer, nearly half of *ta*CA enzymes formed insoluble precipitates (Figure 2b). The low solubility of *ta*CA has been previously reported and was alleviated by increasing the ionic strength of buffer that may screen the attractive interactions between *ta*CA molecules [9]. *ta*CA is a basic protein with an isoelectric point (pI) value of 9.1 and shows highly positive electrostatic surface potential, which is in sharp contrast to *td*CA that has pI value of 6.5 and moderately charged surface potential (Figure 3). According to a computational analysis on the relationship between electrostatic surface properties and protein solubility [30], it appears that the positively charged surface patches of *ta*CA (Figure 3) contribute to the low solubility, although a convincing biochemical explanation has not been provided. In contrast, *td*CA showed no precipitation after purification and dialysis (Figure 2b), indicating its high solubility. Even after the boiling of soluble enzymes for 1 h, a significant fraction of *td*CA still remained soluble while no soluble protein was observed for *ta*CA (Figure 2b). Because the boiled *td*CA lost almost all of the activity (showing only 0.5% of the initial activity), it is suggested that *td*CA can retain its solubility even after denaturation, followed by irreversible inactivation.

### 2.3. Activity and Stability Comparison of Recombinant CAs

**Figure 4:**
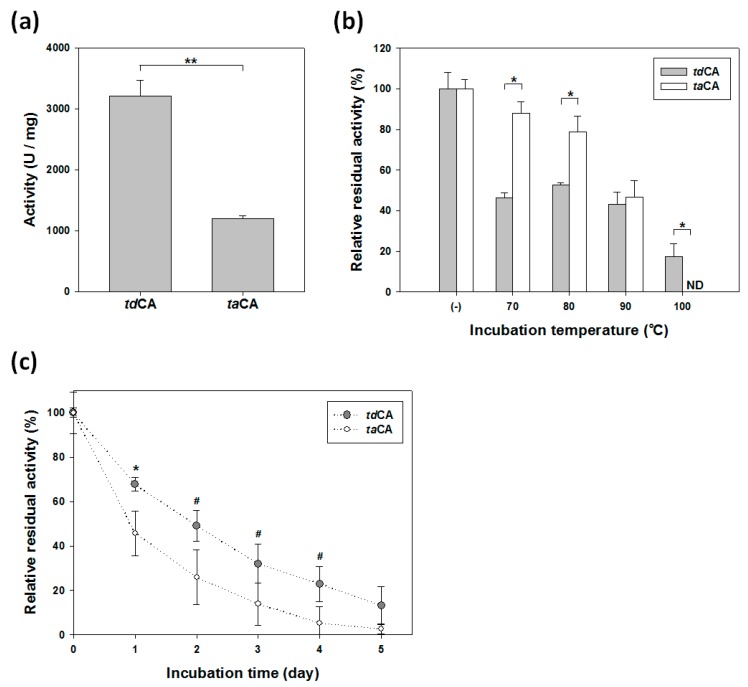
Activities and stabilities of purified *td*CA and *ta*CA. (**a**) CO_2_ hydration activities of the recombinant CAs. (**b**) Short-term stability. The residual activity of each enzyme was measured after 15 min incubation at the indicated temperature. (−), untreated sample. (**c**) Long-term stability at 70 °C. The enzymes were preheated for 15 min at 70 °C and cooled at 4 °C prior to the long-term incubation. Error bars represent standard deviations from two or three independent experiments. Asterisks indicate statistical significance determined by unpaired, two-tailed (for panel **a** and **b**) or one-tailed (for panel **c**) *t*-test (^#^ *p* < 0.1, * *p* < 0.05, ** *p* < 0.01). ND, not detectable.

The purified soluble enzymes were subjected to the activity test. Enzyme activity was measured by an assay that depends on the color change of a pH indicator upon the generation of a proton by CO_2_ hydration reaction. The specific activity of *td*CA (3200 U/mg) was 2.7-fold higher than that of *ta*CA (1200 U/mg) (Figure 4a). Considering the reported high catalytic efficiencies of *ta*CA [9,26], it appears that *td*CA is one of the most efficient catalysts for CO_2_ hydration.

Next, the stability of the enzyme was evaluated by measuring the remaining enzyme activity after heat treatment under high-temperature conditions. The decrease in activity represents the fraction of enzyme inactivated due to the heating [31]. After short-term (15 min) incubation at high temperature ranging from 70 to 90 °C, *td*CA showed similar residual activities (43–53% of its initial activity) regardless of the incubation temperature, while the residual activity of *ta*CA gradually decreased from 88% (at 70 °C) to 47% (at 90 °C) (Figure 4b). At first glance, the stability of *td*CA seemed to be lower than that of *ta*CA because the residual activities of *td*CA were generally lower than those of *ta*CA (Figure 4b). For instance, the residual activity of *td*CA after 15 min incubation at 70 °C was 46%, which was much lower than that (88%) of *ta*CA. At 100 °C, however, *td*CA exhibited 18% residual activity, while *ta*CA completely lost its activity (Figure 4b). These results prompted us to hypothesize that the purified *td*CA enzymes were composed of heat-labile and heat-stable fractions and the sharp decrease of *td*CA in residual activity after the short-term incubation was due to the inactivation of the heat-labile fraction. The remaining heat-stable fraction was assumed to be more stable than *ta*CA. To test this hypothesis, we first incubated the intact enzymes at 70 °C for 15 min, and then, the heat-treated enzymes were taken as the initial samples (0 days) for the subsequent long-term stability test (up to five days) at 70 °C. The result demonstrated that the preheated *td*CA was actually more stable than *ta*CA (Figure 4c). The inactivation showed monophasic, first-order kinetics in both *td*CA and *ta*CA (*R*^2^ > 0.99), implying that the active enzymes from the heat-treated initial sample were composed of a single, heat-stable fraction [32]. The calculated half-life of *td*CA (42.2 h) at 70 °C was 83% longer than that of *ta*CA (23.1 h). It is worth noting that the stability, as well as the solubility of *ta*CA, could be improved by increasing the ionic strength with the addition of 300 mM NaCl [9,20] while the stability of *td*CA was not affected by the addition of salt (data not shown). Collectively, these data show that *td*CA can be a better catalyst than *ta*CA in terms of both activity and stability under general low-salt-containing aqueous conditions.

### 2.4. Engineering of tdCA for High-Level Expression

**Figure 5:**
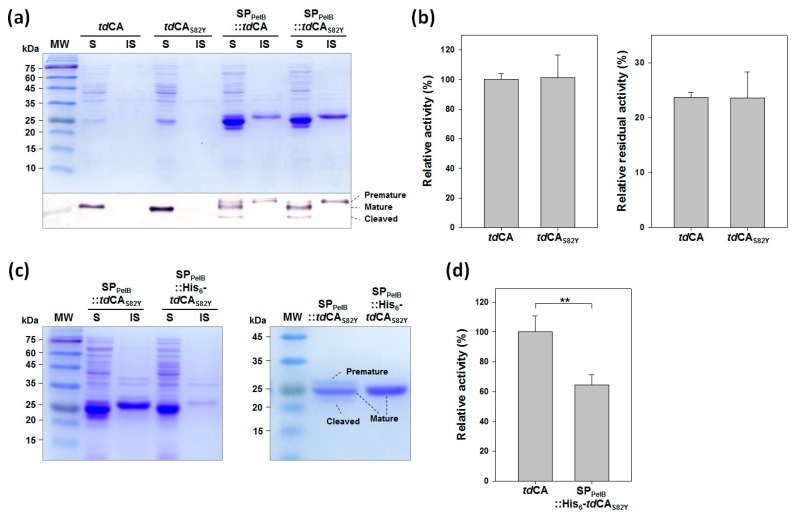
Engineering of *td*CA for high-level expression. (**a**) Expression of *td*CA variants analyzed by SDS-PAGE followed by Coomassie blue staining (upper) and Western blotting using an anti-His_6_ antibody (lower). Cell lysates were fractionated into soluble and insoluble fractions, and they were separately loaded. The samples for the periplasmic CAs were loaded after a 10-fold dilution for Western blot analysis. Lanes: MW, molecular mass marker; S, soluble fraction; IS, insoluble fraction. (**b**) Effect of S82Y mutation on activity (left) and stability (right) of *td*CA. The residual activity of each enzyme was measured after 24 h incubation at 80 °C for the stability test. (**c**) Expression (left) and purification (right) of periplasmic *td*CA_S82Y_ variants with His_6_-tag located at the different terminus. (**d**) Relative activity of periplasmic *td*CA_S82Y_ with N-terminal His_6_-tag compared with that of wild-type *td*CA. Error bars represent standard deviations from two or three independent experiments. Asterisks indicate statistical significance determined by unpaired, two-tailed *t*-test (** *p* < 0.01).

As previously shown, the expression level of *td*CA was relatively low (Figure 2a), which necessitates further improvement of the expression level for the practical applications. In our efforts to improve the stability of *td*CA by protein engineering, we incidentally found that the expression level of *td*CA was significantly increased by the mutation S82Y (Figure 5a). The variant *td*CA_S82Y_ did not show any difference in both activity and stability compared to the wild-type *td*CA (Figure 5b), implying that the mutation S82Y is neutral for the characteristics of *td*CA. Next, we tried to express and translocate *td*CA into the periplasm of *E. coli* via the Sec-dependent pathway, which is a better pathway than the Twin-arginine translocation pathway for CA secretion [33], by genetically fusing PelB signal peptide (SP_PelB_) to the N terminus of *td*CA. This design was originally intended to construct a whole-cell biocatalyst with periplasmic *td*CA (see below). The expression level of SP_PelB_::*td*CA was remarkably improved compared to that of the wild-type, showing a thick band in the SDS-PAGE analysis (Figure 5a). Thus, the periplasmic expression may be used as an alternative strategy for improving the expression level of recombinant protein poorly expressed in the cytoplasm. When combined with the S82Y mutation, the total SP_PelB_::*td*CA_S82Y_, including the insoluble premature form, showed higher expression level compared to that of SP_PelB_::*td*CA, although the expression levels of mature forms were similar to each other (Figure 5a). It appeared that the excess amount of SP_PelB_::*td*CA_S82Y_ was accumulated as an aggregate in the cytoplasm without the cleavage of signal peptide due to the saturation of the Sec-translocon capacity [34]. Consequently, the expression level of mature form from SP_PelB_::*td*CA_S82Y_ increased 8.3-fold compared to that from wild-type *td*CA according to the densitometric analysis on the Western blot result. This result can successfully lead to the reduction of the enzyme production cost.

A minor band for a protein with a smaller molecular weight compared to that of the mature form was observed in the gel electrophoretic analysis when SP_PelB_::*td*CA_S82Y_ (or SP_PelB_::*td*CA) was expressed (Figure 5a). The truncated (or cleaved) form of *td*CA_S82Y_, along with the premature *td*CA_S82Y_ in the soluble faction, was co-purified with the mature *td*CA_S82Y_ upon His_6_-tag affinity purification (Figure 5c). Because the heterogeneity and the low purity of purified enzyme sample might interfere with further biochemical characterization, the His_6_-tag was relocated to the N terminus of *td*CA_S82Y_ (SP_PelB_::His_6_-*td*CA_S82Y_) to exclude the cleaved form of *td*CA_S82Y_ from the affinity purification. Interestingly, the expression analysis of SP_PelB_::His_6_-*td*CA_S82Y_ showed no clear band for the cleaved form or the premature form of *td*CA_S82Y_ (Figure 5c). The N-terminal sequence following the signal peptide appeared to be critical for the efficient periplasmic translocation without the undesirable cleavage. As a result, SP_PelB_::His_6_-*td*CA_S82Y_ was purified to apparent homogeneity (Figure 5c). Unfortunately, the specific activity of SP_PelB_::His_6_-*td*CA_S82Y_ was only 65% of that of wild-type *td*CA (Figure 5d). The change of amino acid sequences of *td*CA_S82Y_ at both N- and C-termini upon the relocation of His_6_-tag might slightly alter the conformation of the enzyme, leading to the activity change. Therefore, although SP_PelB_::His_6_-*td*CA_S82Y_ showed high-level expression and allowed high-purity purification, it seems not to be adequate for the practical applications for CCU when compared to SP_PelB_::*td*CA_S82Y_. It might be useful for other specialized purposes, e.g., protein crystallization requiring a large amount of highly purified protein.

### 2.5. Ultra-Efficient Whole-Cell Biocatalysts Based on tdCA

**Figure 6:**
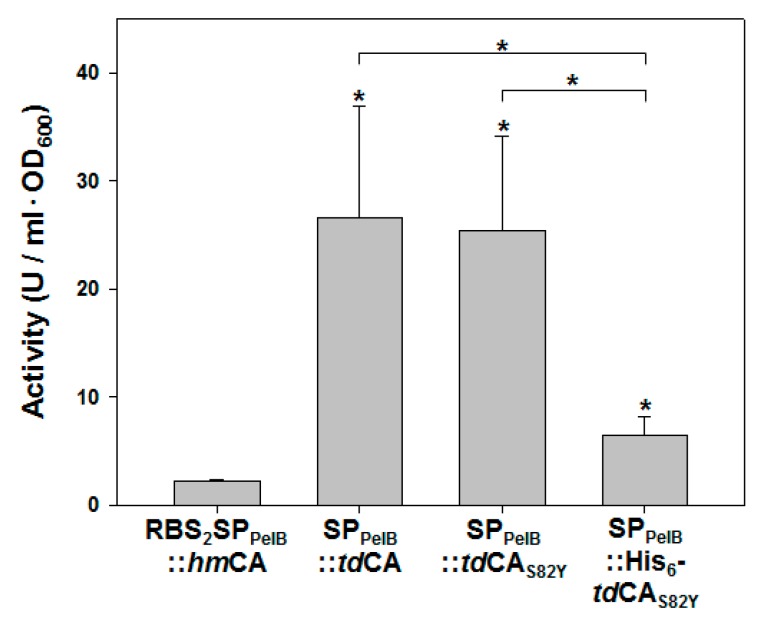
Whole-cell activities of recombinant strains with periplasmic CAs. Any enzymatic activity in the supernatant after centrifugation of the whole-cell solution was subtracted from the total activity to obtain pure whole-cell activity. Error bars represent standard deviations from three independent experiments. Asterisks indicate statistical significance determined by unpaired, two-tailed *t*-test (* *p* < 0.05). An asterisk right above a bar indicates statistical significance compared with RBS_2_SP_PelB_::*hm*CA sample.

Finally, the whole-cell CO_2_ hydration activities of the recombinant *E. coli* strains with highly expressed periplasmic *td*CA were measured to examine the potential of the strains as whole-cell biocatalysts. The whole-cell catalyst with periplasmic *ta*CA was not considered for further testing because the expression level of periplasmic *ta*CA was too low to be compared with that of periplasmic *td*CA (data not shown). Instead, the highly active periplasmic whole-cell biocatalyst (RBS_2_SP_PelB_::*hm*CA) previously constructed by using a halophilic CA (*hm*CA) from *Hydrogenovibrio marinus* and by engineering the ribosome binding site (RBS) was used for the comparison [33]. The strains with periplasmic *td*CA showed much higher whole-cell activities than that of the RBS_2_SP_PelB_::*hm*CA strain (Figure 6). Notably, the activity (26.6 U/mL·OD_600_) of SP_PelB_::*td*CA strain, which was similar to that (25.4 U/mL·OD_600_) of the SP_PelB_::*td*CA_S82Y_ strain, was 11.9-fold higher compared to that (2.2 U/mL·OD_600_) of RBS_2_SP_PelB_::*hm*CA. Although the activity (6.5 U/mL·OD_600_) of the SP_PelB_::His_6_-*td*CA_S82Y_ strain was much lower than that of the SP_PelB_::*td*CA or SP_PelB_::*td*CA_S82Y_ strain, it was still three-fold higher than that of the RBS_2_SP_PelB_::*hm*CA. Considering the reported *k*_cat_ values of *ta*CA (9.6 × 10^5^ s^−1^–1.6 × 10^6^ s^−1^) [9,26] and *hm*CA (3.3 × 10^5^ s^−1^) [12], the high activity of *td*CA enzyme (Figure 4a) seems to be the primary factor that contributes to the remarkably high activity of the *td*CA-based whole-cell biocatalysts. Thus, the engineered strains with highly expressed periplasmic *td*CA could be used as ultra-efficient CO_2_-capturing whole-cell biocatalysts.

## 3. Materials and Methods 

### 3.1. General Culture Conditions, Bacterial Strains, and Plasmids Construction

The *E. coli* strains, plasmids, and primers used in this study are listed in Table 1. *E. coli* TOP10 was used for the DNA works, and *E. coli* BL21(DE3) strain was used for recombinant protein expression. Luria-Bertani (LB) medium supplemented with antibiotics was used for the culture at 37 °C and 220 rpm in a shaking incubator (Jeiotech, Daejeon, Korea). Fifty μg/mL ampicillin or 10 μg/mL streptomycin was supplemented to the culture media of recombinant strains or wild-type *E. coli* TOP10, respectively. The *td*CA gene (GenBank accession number: OAQ21602) was chemically synthesized with codon optimization for *E. coli* (Genscript, Piscataway, NJ, USA) with a codon adaptation index (CAI) of 0.96 and was subcloned into pET-22b (+) (Novagen, Madison, WI, USA) using *Nde*I and *Xho*I restriction sites, resulting in pET-tdCA. The putative signal peptide of 20 amino acids was not included in the recombinant *td*CA. The previously constructed *ta*CA gene was also codon-optimized with a CAI of 0.88 [9]. For the mutation of S82Y, one-step polymerase chain reaction (PCR)-based mutagenesis was performed [35] using pET-tdCA as the template plasmid using the listed primers, resulting in pET-tdCA-S82Y. For the periplasmic expression, the *td*CA gene was amplified by PCR and was subcloned into pET-22b(+) using *Nco*I and *Xho*I restriction sites, resulting in pET-PelB-ss::tdCA, pET-PelB-ss::tdCA-S82Y or pET-PelB-ss::Nhis_tdCA-S82Y. The recombinant *td*CA derivatives except for SP_PelB_::His_6_-*td*CA_S82Y_ have a His_6_-tag sequence at the C terminus.

### 3.2. Expression of Recombinant CA Enzymes

*E. coli* BL21(DE3) strains transformed with the recombinant plasmids were incubated in LB medium at 37 °C and 180 rpm in the shaking incubator. At an OD_600_ of 0.6–0.8 measured using a UV-Vis spectrophotometer (Shimadzu, Kyoto, Japan), the medium was supplemented with 1 mM isopropyl-β-D-thiogalactopyranoside (Duchefa Biochemie, Haarlem, Netherlands) and 0.1 mM ZnSO_4_ (Junsei, Tokyo, Japan) for the induction of recombinant protein expression. The cells were further cultivated for 12 h at 37 °C and 180 rpm, harvested by centrifugation at 4 °C and 4000× *g* for 10 min, and resuspended in lysis buffer (50 mM sodium phosphate, 300 mM NaCl, and 10 mM imidazole; pH 8.0). The cells were lysed by an ultrasonic dismembrator (Sonics and Materials, Newtown, CT, USA) for 20 min on ice water. The lysate was centrifuged at 4 °C and 10,000× *g* for 10 min. The pellets were designated the insoluble fraction (IS) and the supernatants were designated the soluble fraction (S).

### 3.3. Purification of Recombinant CA Enzymes.

Prior to affinity purification of target enzymes, heat-labile endogenous host proteins were heat-precipitated by incubating the lysates at 60 °C for 20 min. After centrifugation of the heat-treated lysates at 4 °C and 10,000× *g* for 10 min, the supernatants were mixed with Ni^2+^-nitrilotriacetic acid agarose beads (Qiagen, Germantown, MD, USA), and the His_6_-tagged target proteins were purified according to the manufacturer’s instructions. The enzymes were eluted using elution buffer (50 mM sodium phosphate, 300 mM NaCl, and 250 mM imidazole; pH 8.0). The purified recombinant CAs were thoroughly dialyzed against 20 mM sodium phosphate buffer (pH 7.5) at 4 °C. In the case of *ta*CA, precipitated enzyme after dialysis was removed by centrifugation at 4 °C and 10,000× *g* for 10 min, and the soluble enzymes in the supernatant were used for further biochemical analyses. The enzyme concentrations were adjusted to 5 µM before the experiments.

### 3.4. Protein Quantification

The purified enzyme was denatured in denaturing buffer (6 M guanidine hydrochloride GuHCl/20 mM sodium phosphate; pH 7.5), and the absorbance of the denatured protein was measured at 280 nm in a quartz crystal cuvette. The concentration of the purified protein was determined using the measured absorbance, and the calculated extinction coefficient at 280 nm by ProtParam (http://web.expasy.org/protparam/) [36].

### 3.5. SDS-PAGE and Western Blot

Protein samples were separated by sodium dodecyl sulfate-polyacrylamide gel electrophoresis (SDS-PAGE) and visualized by Coomassie blue R-250 (Bio-Rad, USA) staining. For Western blotting, the separated proteins were blotted onto a nitrocellulose membrane (Whatman, Clifton, NJ, USA). Monoclonal anti-His_6_ antibody (ABM, Canada) and alkaline phosphatase-conjugated anti-mouse immunoglobulin G (Bethyl Laboratories, Montgomery, TX, USA) were sequentially treated. The His_6_-tagged target proteins were visualized by color development using the substrate nitroblue tetrazolium–5-bromo-4-chloro-3-indolyl phosphate (NBT/BCIP; Sigma-Aldrich, St. Louis, MO, USA).

### 3.6. CO_2_ Hydration Assay

The CO_2_ hydration activity was assayed by a colorimetric method [12,37]. 10 or 20 μL of the sample was added to the disposable cuvette containing 600 μL of 20 mM Tris buffer (pH 8.3) supplemented with 100 μM phenol red. The reaction was performed at 4 °C inside the spectrometer by adding 400 μL of CO_2_-saturated deionized water prepared in ice-cold water. The absorbance change was monitored at 570 nm. The time (*t*) required for the absorbance to decrease from 1.2 (corresponding to pH 7.5) to 0.18 (corresponding to pH 6.5) was determined. The time (*t*_0_) for the uncatalyzed reaction was also measured by adding a corresponding blank buffer instead of an enzyme sample. The enzyme unit (U) was calculated, as (*t*_0_ − *t*) / (*t* × 5) as previously described [33].

### 3.7. Thermostability Test

The samples were incubated at the indicated temperatures for the appropriate time and then immediately cooled on ice. The activities of the incubated samples were measured and compared with the activities of the non-incubated samples. The residual activities were calculated and presented as relative residual activity (%).

### 3.8. Whole-cell Activity

The whole-cell activity was measured as previously described [33]. After cultivation, cells were harvested and resuspended in phosphate-buffered saline (PBS; 8 g/l NaCl, 0.2 g/l KCl, 1.44 g/l Na_2_HPO_4_, and 0.24 g/l KH_2_PO_4_) at a cell concentration of 8–14 OD_600_. For whole-cell activity measurement, the obtained kinetic data after CO_2_ hydration assay were corrected by subtracting the absorbance of the whole-cell from the measured absorbance at 570 nm. Any activity of leaked enzyme from the prepared cells was estimated by measuring the activity of supernatant after the centrifugal removal of the cells from the cell suspension and was subtracted from the total activity to obtain the pure whole-cell activity.

### 3.9. In Silico Calculations

The multiple sequence alignment was performed using ClustalX 2.0, and the aligned sequences were shaded with Boxshade 3.21 (https://embnet.vital-it.ch/software/BOX_form.html). The three-dimensional structure of *td*CA was constructed by protein threading modeling using I-TASSER [38]. The best threading templates used for the construction included CAs from *Neisseria gonorrhoeae* (PDB ID: 1KOP), *Sulfurihydrogenibium azorense* (PDB ID: 4X5S), and *Thermovibrio ammonificans* (PDB ID: 4C3T, 4COQ). The calculation and visualization of the surface electrostatic potential were performed on UCSF Chimera using Coulombic surface coloring [39]. Signal peptide cleavage sites were predicted by the SignalP 5.0 server (http://www.cbs.dtu.dk/services/SignalP/) [40]. Densitometric analysis of the protein band on Western blot was performed using ImageJ [41].

## 4. Conclusions

The recombinant *td*CA was successfully expressed and purified in *E. coli* BL21(DE3). The solubility of *td*CA was higher than *ta*CA. The CO_2_ hydration activity of purified *td*CA was 2.7-fold higher than that of *ta*CA. The purified *td*CA enzymes appeared to be composed of heat-labile and heat-stable fractions. Although *td*CA easily lost a fraction of activity by short-term heat treatment due to the inactivation of heat-labile fraction, the higher stability of the remaining heat-stable fraction and the higher activity make *td*CA a better catalyst compared to *ta*CA under low-salt aqueous solutions. The relatively low expression level of *td*CA was overcome by the S82Y mutation and the periplasmic secretion via the Sec-dependent pathway. The combined strategy resulted in at least an 8.3-fold higher expression level of *td*CA compared to that of wild-type *td*CA. In addition, the *E. coli* cells with the periplasmic *td*CA variant displayed 11.9-fold higher whole-cell activity compared to that of the recently reported system with a halophilic CA. These results demonstrate that the highly expressed periplasmic *td*CA can be used as a promising biocatalyst for CCU applications under low-salt conditions.

## Figures and Tables

**Table 1:** Strains, plasmids, and oligonucleotide primers used in this study.

Strains, Plasmids, or Primers	Genotypes, Relevant Characteristics, or Sequences	Source or References
Strains		
*E. coli* TOP10	F^−^ *mcrA* Δ*(mrr-hsdRMS-mcrBC)* Ф80*lacZ*ΔM15 Δ*lacX*74 *recA*1 *araD*139 Δ*(ara-leu)*7697 *galU galK rpsL*(Str^r^) *endA*1 *nupG*	Thermo Fisher Scientific
*E. coli* BL21(DE3)	F^−^ *ompT hsdS*_B_(r_B_^−^ m_B_^−^) *gal dcm lon* λ(DE3), carrying T7 RNA polymerase gene	Novagen
Plasmids		
pET-22b(+)	T7*lac* promoter, pBR322 *ori*, Amp^r^, parental expression vector	Novagen
pET-taCA	pET-22b(+) carrying *ta*CA gene	[9]
pET-tdCA	pET-22b(+) carrying *td*CA gene	This study
pET-tdCA-S82Y	pET-22b(+) carrying *td*CA_S82Y_ gene	This study
pET-PelB-ss::tdCA	pET-22b(+) carrying SP_PelB_::*td*CA gene	This study
pET-PelB-ss::tdCA-S82Y	pET-22b(+) carrying SP_PelB_::*td*CA_S82Y_ gene	This study
pET-PelB-ss::Nhis_tdCA-S82Y	pET-22b(+) carrying SP_PelB_::His_6_-*td*CA_S82Y_ gene	This study
pRBS_2_PelB-ss::hmCA	pET-22b(+) carrying periplasmic *hm*CA gene and a mutant RBS	[33]
Primers ^1^		
tdCA-S82Ymut	Forward: TAACTTTCACTACCGTGACCAAATCTATGGCGAGATTGTGAACAACG	This study
	Reverse: CGTTGTTCACAATCTCGCCATAGATTTGGTCACGGTAGTGAAAGTTA	
sec-tdCA	Forward: CCATGGGTGGCGGTCA	This study
	Reverse: CTCGAGTTTCAGAATCTTACGCGCG	
sec-Nhis_tdCA	Forward: CCATGGGCAGCAGCCATCATCATCATCATCACAGCAGCGGTGGCGGTCACGT	This study
	Reverse: CTCGAGTTATTTCAGAATCTTACGCGCG	

^1^ Restriction sites are underlined, and the mutated regions are indicated in bold.
